# Dual-Functional On-Chip AlGaAs/GaAs Schottky Diode for RF Power Detection and Low-Power Rectenna Applications

**DOI:** 10.3390/s110808127

**Published:** 2011-08-18

**Authors:** Abdul Manaf Hashim, Farahiyah Mustafa, Shaharin Fadzli Abd Rahman, Abdul Rahim Abdul Rahman

**Affiliations:** 1 Material Innovations and Nanoelectronics Research Group, Faculty of Electrical Engineering, Universiti Teknologi Malaysia, 81310 Skudai, Johor, Malaysia; E-Mails: farzies@gmail.com (F.M.); shaharinfadzli@fke.utm.my (S.F.A.R.); rahim@fke.utm.my (A.R.A.R.); 2 Ibnu Sina Institute for Fundamental Science Studies, Universiti Teknologi Malaysia, 81310 Skudai, Johor, Malaysia

**Keywords:** AlGaAs/GaAs, HEMT, Schottky diode, RF power detector, rectenna

## Abstract

A Schottky diode has been designed and fabricated on an *n*-AlGaAs/GaAs high-electron-mobility-transistor (HEMT) structure. Current-voltage (*I–V*) measurements show good device rectification, with a Schottky barrier height of 0.4349 eV for Ni/Au metallization. The differences between the Schottky barrier height and the theoretical value (1.443 eV) are due to the fabrication process and smaller contact area. The RF signals up to 1 GHz are rectified well by the fabricated Schottky diode and a stable DC output voltage is obtained. The increment ratio of output voltage *vs* input power is 0.2 V/dBm for all tested frequencies, which is considered good enough for RF power detection. Power conversion efficiency up to 50% is obtained at frequency of 1 GHz and input power of 20 dBm with series connection between diode and load, which also shows the device’s good potential as a rectenna device with further improvement. The fabricated *n*-AlGaAs/GaAs Schottky diode thus provides a conduit for breakthrough designs for RF power detectors, as well as ultra-low power on-chip rectenna device technology to be integrated in nanosystems.

## 1. Introduction

Ubiquitous Network Society is an umbrella term for contemporary visions about the future of information technology [1]. A so called Intelligent Quantum (IQ) chip with sizes of several millimeters square proposed by Hasegawa *et al.* [2] could be part of this ubiquitous concept. An IQ chip is an III–V semiconductor chip where nanometer scale quantum processors and memories are integrated on the same chip with other capabilities such as wireless power supply, detector, optical devices and various sensing functions. Ideally, such a III–V semiconductor chip should be placed on a Si platform. Recently, we reported the proposal of direct integration of an antenna and a Schottky diode for a RF power detector as well as a rectenna (rectified antenna) device application to supply direct current (DC) power to generate other on-chip nanodevices [3,4].

Nanoelectronic systems are increasingly vulnerable to malfunctions due to incident electromagnetic (EM) radiation, particularly since many integrated circuits operate at lower and lower voltages. The damaging RF radiation can be produced intentionally, such as by a high power microwave generator [5], or accidentally, such as by ambient sources like lightning. Thus, it is of great interest to know how, and at what level, microwaves penetrate equipment shielding and reach the vulnerable chips. This has motivated our group to work on on-chip RF detectors both for measuring power at the chip level and for developing strategies to mitigate its effects. Knowing the RF power levels in various chips and locations within chips is likely to be more useful than the “digital” information that a given external RF power level made the circuits fail.

Schottky diodes are known as fast rectifying devices and can be used as RF detectors [6]. In special molecular beam epitaxy (MBE) grown geometries, RF detection up to 100 GHz has been reported [7]. However, in foundry fabricated Si-based diodes detection of only up to 600 MHz has been reported [8]. Recently, CMOS fabricated Schottky diode-detected RF signals up to 10 GHz in direct injection experiments and in the range of 9.5–19.5 GHz in microwave irradiation experiments have also been reported [9]. However, to our knowledge there is no report on the design and fabrication of n-AlGaAs/GaAs HEMT Schottky diode for RF power detection.

Low-power rectenna devices are among the most promising devices to be integrated on the IQ chip to form the wireless power supply. Basically, the rectenna should have small dimensions as well. Because of this requirement, wireless power transfer using such a device is considered to be suitable for low power applications. To our knowledge, almost all past rectennas were designed for over 100 milliwatt (mW) rectifying and the RF-to-DC power conversion efficiency is less than 20% at the 1 mW microwave input level [10]. Various kinds of rectennas today have been developed by integrating discrete diodes and antennas through matching circuits. Consequently, the dimensions of the rectennas increase; this results in a high cost device. Thus, small dimension rectenna devices need to be developed. In this study, we propose a dual-functional on-chip AlGaAs/GaAs Schottky diode for RF power detector and low-power rectenna applications where a diode is equipped with a coplanar waveguide (CPW) transmission line for realizing direct integration without insertion of any matching circuit.

As a semiconductor material for Schottky diode, III–V-based compound materials have been considered the most promising materials because of their stability, capability of making a good Schottky contact and well-developed fabrication process technology. Higher electron mobility exists in two dimensional electron gas (2DEG) layer making it suitable for high-frequency devices up to terahertz (THz) range [11–19]. They have also emerged to be suitable for nanostructure formation for the development of the IQ chip [2].

However, the design and fabrication of planar dipole antennas and Schottky diodes on III–V semiconductor based HEMT structures for RF power detectors and rectennas have not been extensively investigated. In this study, the design and fabrication of a Schottky diode on an *n*-AlGaAs/GaAs HEMT structure is further reported. The output voltages and RF-DC power conversion efficiency of the Schottky diode are evaluated and discussed, which seem to raise hope towards realizing an on-chip RF power detector as well as an ultra-low DC power supply to generate other integrated on-chip nanodevices.

## 2. Importance of GaAs Based Chip Structure on Si Platform and Its Technical Challenges

The evaluation of the GaAs Schottky diode is the main purpose of this study. Therefore, the fabrication and characterization is directly done on the AlGaAs/GaAs substrate. However, to make our motivation for this work clear to the readers, it is important to briefly discuss the importance of GaAs on Si structures and their technical challenges in the growth technology. It is well known that the performance of Si-LSIs has been enhanced over the last 30 years by increasing the number of transistors according to Moore’s law [20]. The number of transistors in the latest processor is already over 1 billion [21]. As is well known, the scaling rule of the Si transistor has made it possible to enhance the performance of the LSIs. However, the miniaturization of the transistors becomes increasingly difficult due to the physical limitations, and the conventional scaling rule will not be enough to enhance the performance of the LSIs. Therefore, some breakthrough technologies are strongly required for the Si LSI in order to enhance the device and system performance, even in the post-scaling era. For example, to date, strained Si [22], high-k gate insulator/metal [23], *etc.* have been applied to increase the performance of CMOS. However, in the gate length regime of 16 nm, the driving current by strained Si cannot be increased further.

The most promising breakthrough technology is the introduction of new semiconductor materials with higher mobility than Si which can increase the driving current of the MOS transistor. III–V compound semiconductors are among the most promising candidates for channel materials [24]. These materials are also expected to give high carrier injection velocity at the source terminal due to low effective mass and low carrier scattering, which leads to an increase in the efficiency of ballistic transport. In addition, these materials are also applicable to photonic and sensing devices. Therefore, the combination of this material on Si should lead to the realization of the so-called Heterogeneous Integration (integration between various functional devices and CMOS), hence leading to the realization of IQ chip. Ideally the III–V materials need to be realized on the Si platform, meaning that they should be grown on large-area Si wafer, compatible with standard Si CMOS fabrication technology and also Si CMOS LSI design environment.

For several years, there has been a great deal of activity in the growth of GaAs compounds on Si substrates [25]. The major reason for such activity is because of the high quality, low cost and large wafer size offered by Si substrates. The GaAs substrate is much more expensive than Si and the wafer size is still considerably small. Presently, 4-in. is the maximum GaAs wafer diameter in comparison to the current 10-in. Si wafer sizes. The growth of GaAs on such Si wafers is an advantageous way of growing GaAs on large-diameter wafers. The most exciting motivation for GaAs on Si, is the hybridization of Si and GaAs technologies on the same wafer. GaAs and Si technology are highly complementary and compatible. The advantage of Si over GaAs is its highly developed processing technology and the very high integration density of circuits available. The advantage of GaAs over Si is its intrinsically higher performance potential and its optical properties. By integrating the GaAs-based MOSFETs on Si CMOS platform, not only can the capability and functionality be increased, but this will also increase the value of device systems. The introduction of GaAs channels can enhance the driving current of CMOS devices. In addition, since GaAs has higher electron mobility than Si, this material can be also used for conventional n-MOS. Thus they have the potential in enabling future high-speed transistors for digital applications at very low supply voltages. In other word, the present Moore’s Law can be further extended. This technology is commonly known as “More Moore” technology.

GaAs can not only be used to fabricate conventional MOSFET but also other type of MOSFET devices with different switching principles such as plasma wave FET [13,26], ballistic FET [27], tunnel FET [28] and spin FET [28]. Such directions lead to the “Beyond CMOS” technology. GaAs can also be used to fabricate other functional devices such as RF detectors and rectennas (ex: this work), optical devices and sensors to be integrated with conventional Si CMOS and other Si based functional devices such RF-CMOS, NEMS etc to realize the IQ chip concept [29]. Therefore, the value of hybridization of GaAs and Si technologies is apparent. One can imagine such a single chip where the high speed devices and optical inputs and outputs are driven by GaAs devices and the highly integrated parts of the circuits are implemented in Si technology.

However, direct growth of GaAs-on-Si is particularly challenging, due to the large lattice mismatch and the polar/non-polar nature of the GaAs system, which generate high densities of threading dislocations and anti-phase domains (APDs), respectively [30]. Fortunately, the growth of high quality GaAs on Si should be possible with the introduction of a Ge buffer layer [31]. Ge is an ideal intermediary material between GaAs and Si because it is both lattice matched to GaAs and compatible with the Si technology, and recently, has drawn considerable attention for the direct growth of GaAs-on-Si [32]. Moreover, a germanium-on-insulator-on-silicon (GeOI) substrate has been proposed as a potential platform for the monolithic integration of GaAs devices with complementary metal-oxide-semiconductor technology [32]. In addition, since Ge has higher hole mobility than Si, this material can be also used for conventional p-MOS to be co-integrated with GaAs n-MOS.

It is clear that GaAs on Si based hetero-integration chip has good potential to be commercially available in the near future. The fundamental work presented in this paper on GaAs based Schottky diodes should contribute to the development of GaAs RF power detectors and low-power rectenna devices which are considered as ideal devices for directly integration on chips to form wireless power supplies.

## 3. Design and Fabrication of the Schottky Diode

The *n*-AlGaAs/GaAs HEMT structure was chosen because of the higher electron mobility that can be provided by the 2DEG layer. The sample is an AlGaAs/GaAs modulation-doped heterostructure grown by molecular beam epitaxy. The interface of *n*-doped AlGaAs layer and undoped GaAs layer defines a 2DEG system where electron motion perpendicular to the layer is frozen out, thus producing highly mobile electrons. Higher electron mobility thus exists in a 2DEG layer, making it suitable for high speed and high-frequency devices. This unique feature formed by AlGaAs/GaAs HEMT structures make them suitable as a core material for the development of the IQ chip which has been considered as the most promising chip structure for the future ubiquitous network society.

**Figure 1.**
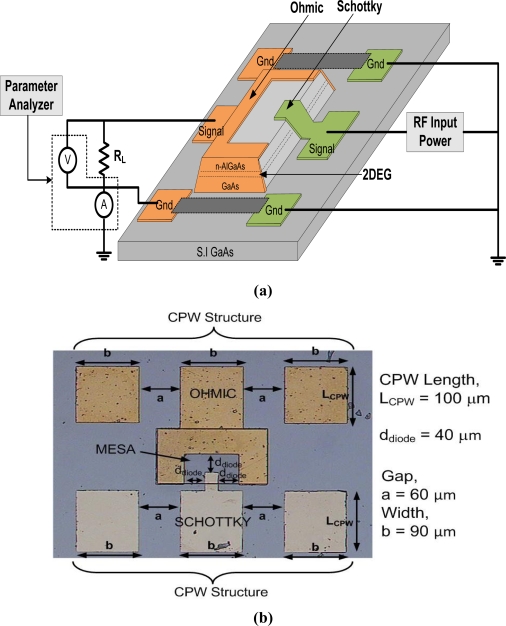
**(a)** Schematic of device structure (including measurement circuit) and **(b)** fabricated Schottky diode (photo of top view).

Figure 1(a) shows the schematic of device structure including the applied RF measurement circuit. The layer and thickness of *n*-AlGaAs/GaAs HEMT structure, from bottom to top are as follows: 625 μm semi-insulated high-dielectric constant GaAs substrate, 500 nm GaAs buffer layer; 100 nm AlGaAs buffer layer; 20 nm undoped GaAs layer; 10 nm AlGaAs spacer layer; 50 nm *n*-doped AlGaAs (Si δ (delta) doping) barrier layer; 10 nm *n*-GaAs cap layer. The carrier mobility and the carrier sheet density obtained by Hall measurements at room temperature are 6040 cm^2^/V-sec and 8.34 × 10^11^ cm^−2^, respectively. In this work, high doping concentration of n-GaAs cap layer (1 × 10^18^ cm^−3^) and n-AlGaAs/GaAs barrier layer (3.0 × 10^12^ cm^−3^) were selected so that high carrier sheet density of two-dimensional electron gas can be realized. In addition, such high doping concentration of cap layer can produce good ohmic contacts for the devices. The effect of doping concentration of the n-GaAs cap layer on the SBH is not studied in this work. However, it is expected that the SBH should decrease with the increase of doping concentration. Hudait *et al.* [33] reported the doping dependence of the SBH in the concentration range of 2.5 × 10^−5^ − 1.0 × 10^18^ cm^−3^. The significant decrease in barrier height at high doping concentrations can be explained by thermionic field-emission theory [33].

As shown in Figure 1(a), our devices are equipped with a coplanar waveguide (CPW) transmission line on both sides of the Schottky and ohmic contacts which possess ground-signal-ground (G-S-G) pad structures. The dimension of the gap, *a* and width, *b* for CPW determined by the Wheeler’s equation [34] are chosen to be 60 μm and 90 μm, respectively, in order to produce the characteristic impedance, *Z_o_* of 50 Ω. This CPW dimensions are similar to the proposed antenna structures [3] where it can make the direct integration without insertion of matching circuit possible. The CPW structure also permits direct injection of RF signal through Cascade G-S-G Infinity-150 microprobers.

In this preliminary study, the Schottky contact area, *A* is 20 μm × 20 μm, the lengths of CPW, *L_CPW_* is 100 μm and the distance between Schottky-ohmic contacts, *d_diode_* is 40 μm. To achieve high cut-off frequency, the Schottky contact area needs to be small since the cut-off frequency of Schottky diode increases with the decrease of contact area. The detail discussion on the cut-off frequency of the fabricated diode can be found in [3]. Shorter CPW length is chosen in order to omit any matching circuit for the direct integration. Besides that, it also can reduce specific area for making it a low cost rectenna. Longer CPW length will affect the resonant frequency of our proposed antenna based on our separate pre-simulation results on the integrated CPW-dipole antenna analysis using the commercial Electromagnetic Sonnet Suites simulator. We have presented the design and RF characteristics of planar dipole antenna facilitated with CPW structure for possible integration with Schottky diode [3]. The aim is to realize a rectenna device by integrating a dipole antenna and Schottky diode through a CPW transmission line without any insertion of matching circuit in between [3].

The Schottky diodes are patterned and fabricated using photolithography and a standard lift-off technique. The processing steps used in the fabrication are the conventional steps used in a standard GaAs processing. The mesa or channel structure is formed by wet etching method (H_2_SO_4_:H_2_O_2_:H_2_O; 8:1:1). The ohmic contact is realized by Ge/Au/Ni/Au alloy which is annealed at 430 °C for 5 min in N_2_ ambient. The Schottky contact is formed by Ni/Au. The depositions of contact metals are done at pressure of 5 × 10^−6^ Torr using electron beam evaporator. A photo of the as-fabricated Schottky diode is shown in Figure 1(b).

## 4. Results and Discussion

### 4.1. DC Current-Voltage (I–V) Measurement

**Figure 2.**
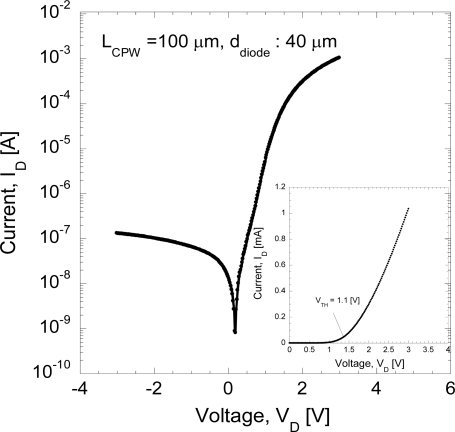
DC *I–V* curve of fabricated Schottky diode.

Figure 2 shows the DC *I–V* characteristics measured using a Keithley semiconductor characterization system model 4200 and micromanipulator probe station. As shown in Figure 2, the DC *I–V* curve of a fabricated Schottky diode shows a diode *I–V* curve with a 1.37 kΩ total series resistance, *R_s_* defined as the slope between 2 and 3 V. The threshold voltage, *V_th_*, for this device is estimated to be 1.1 V, as shown in Figure 2 (inset). From the DC *I–V* curve, it can be said that good rectification is obtained. Details of the rectification characteristics of similar AlGaAs/GaAs Schottky diode can also be found in [3]. Measurement of the reverse saturation current of the device is used to calculate the Schottky barrier heights (SBHs) by applying the Richardson-Dushman equation for the thermionic current [6]:

(1) $$\varphi_{b}=V_{t}\cdot \text{ln}\left(\frac{A\cdot A^{*}\cdot T^{2}}{I_{s}}\right)$$

where *φ_b_* is the barrier height in volts, *I_s_* is the reverse saturation current, A* is the effective Richardson constant (8.16 Acm^−2^ K^−2^), *A* is the area of the metal-semiconductor contact, *T* is the absolute temperature and *V_t_* is the thermal voltage. The calculated Schottky barrier height is 0.4349 eV. This experimental barrier height is lower than the ideal calculated value of 1.443 eV.

The discrepancy of Schottky barrier height values between the experiment and theory is possibly due to the fabrication process, as suggested by Zhang *et al.* [35]. They reported the characteristics of Schottky contacts of different metals to n-type AlGaAs/GaAs structures. A model, which explains the quality of the contact and defect formation at the semiconductor surface due to interdiffusion and/or penetration of metal to the semiconductor, was proposed. Their model can qualitatively explain the difference in barrier heights and degradation of barrier due to certain processes. In addition, it was also reported by Milanovic *et al.* [8] that the work functions of the metal and the semiconductor are determined by the fabrication process. The actual nature of the metal-semiconductor contact is not controllable and in fact may vary substantially from one process to another. The lowering of Schottky barrier height is also due to a small contact area as this parameter is included in Equation (1). Therefore, the area of the diode, *A* is the major design parameter since most of the other parameters such as the work function of the metal and the semiconductor are determined by the fabrication process and interface properties.

### 4.2. RF Measurements

**Figure 3.**
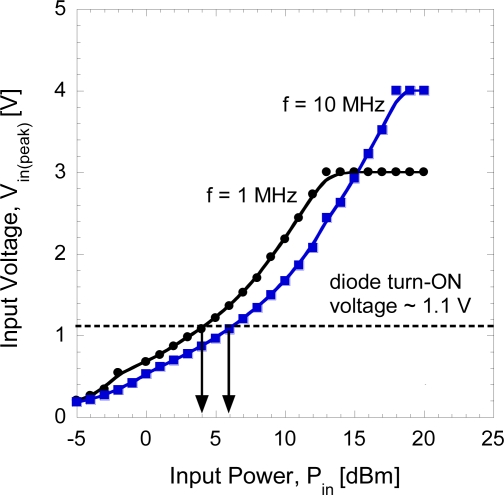
Generated input voltages as a function of injection powers.

First, we investigate the voltages that can be generated by the signal generator (model Agilent 83650B, 10 MHz–50 GHz) in order to confirm the level of voltages at each input power level. In this measurement, an oscilloscope (model: Tektronics TDS 3054C) is connected directly to the signal generator. This is only performed at low frequencies of 1 and 10 MHz in order to confirm the relationship. Figure 3 shows the generated half-peak voltage, *V_in_*_(_*_peak_*_)_ of signal generator as a function of input power, *P_in_*.

**Figure 4.**
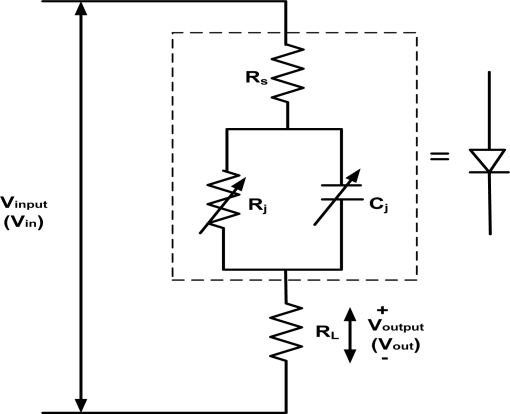
Equivalent model of the measurement setup.

As shown in Figure 3, we need to apply more than 5 dBm of input power at 1 MHz in order to turn the diode on since the turn-on voltage of diode is about 1.1 V. Furthermore, from the equipment characteristics, the following can be concluded: (i) the generated half-peak voltage increases with the input power, but becomes constant after reaching a certain input power level (*i.e*., 12 dBm for 1 MHz and 17 dBm for 10 MHz, 18 dBm for 50 MHz and 20 dBm for 1 GHz; measurements after those points will not be considered), and (ii) it is also noted here that the generated voltage with frequency of 50 MHz and 1 GHz reaches 1.1 V at input powers of 7 dBm and 10 dBm, respectively. The RF characteristics of the Schottky diodes, which have a 20 μm × 20 μm contact area, are evaluated by directly injecting RF power through the G-S-G CPW structure using Cascade Infinity-150 microprober. We assemble a simple measurement setup as shown in Figure 1(a). The equivalent model is shown in Figure 4 where the diode and load are connected in series form. The diode rectifies the incident RF signal and the load produces a DC output. The load resistance, *R_L_* of 50 Ω at the output side is grounded to the RF source. The output voltage is measured at load resistance using parameter analyzer (model: Keithley Semiconductor Characterization System 4200).

**Figure 5.**
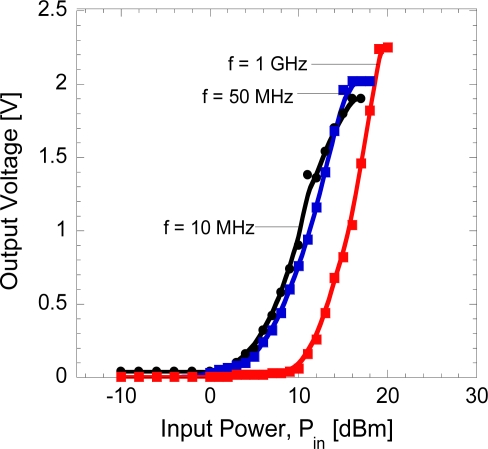
Rectified output voltages as a function of input voltages at frequency of 10 MHz, 50 MHz and 1 GHz.

Figure 5 shows the rectified output voltages as a function of input voltages at different frequency of 10 MHz, 50 MHz and 1 GHz.

It can be seen that the rectified output voltages are only obtainable when the input voltages exceed the turn-on voltage of the diodes which is 1.1 V or 5 dBm for 10 and 50 MHz, and 10 dBm for 1 GHz. The power conversion efficiency of a diode generally changes with the input power. The efficiency is small in the lower power region because the voltage swing at the diode is below or comparable with the forward voltage drop of the diode. The efficiency increases as the input power increases. It was reported in [36] that the efficiency will sharply decrease as the voltage swing at the diode exceeds the breakdown voltage, *V_br_* of the diode. The critical input power where the breakdown effect becomes dominant is expressed as *V_br_*^2^/4*R_L_*. The three parameters of diode, *V_br_*, junction capacitance, *C_j_* and series resistance, *R_s_* determine the power conversion efficiency. We have also shown that the *R_s_* should be small enough to operate at higher frequency with high conversion efficiency [4].

For a small input voltage or power, the series resistance can be ignored because the junction resistance is much bigger than the series resistance. Mathematically, if the thermionic emission is dominant among the carrier transport mechanisms, a diode obeys Equation (1). The Equation (2) can be written as a power series for the better analysis of rectifying action [36,37]:

(2) $$\begin{array}{l} I=I_{s}\left(e^{\frac{q}{\mathrm{nkT}}V}-1\right)\\ \;\;=I_{s}\left(\frac{q}{\mathrm{nk}T}V+\frac{{\left(\frac{q}{\mathrm{nKT}}V\right)}^{2}}{2!}+\frac{{\left(\frac{q}{\mathrm{nkT}}V\right)}^{3}}{3!}+...\right) \end{array}$$

**Figure 6.**
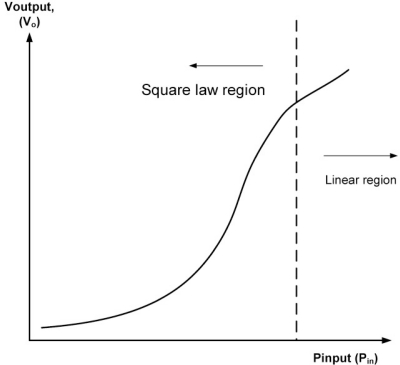
Output voltage *vs.* input power [38].

Here, the second and other even-order terms of this series provide the rectification because negative voltage input becomes positive component in this series. The second order term is the most significant in a small signal operation and dc output is proportional to the square of power level, the diode is said to be operating in the square law region [38]. If the input signal becomes larger, the third term and higher terms become significant and the series resistance cannot be ignored, the diode is no longer in the square law operation region and moves into the linear region where the output voltage is proportional to the input voltage or power, as illustrated in Figure 6. As reported in [38], the square law region can be extended up to 20 dBm by using GaAs semiconductor material.

In general, the tendency shown in Figure 6 has been clearly seen experimentally as presented in Figure 5. However, the major discrepancy is that the linear region is not observed but the output voltage tends to saturate at high input power. Michael *et al.* [39,40] reported that the coupling of micro/nano-scale switching devices with load or parasitic capacitance in series, the resistance blow-up is expected to occur at high electric field which results in an enhanced RC time constant. Greenberg and del Alamo [41] presented direct experimental evidence of resistance blow-up in InGaAs hetero-field-effect transistor (HFET). The similar phenomena may occur in the fabricated diode at input power of 15 dBm (10 MHz), 16 dBm (50 MHz) and 18 dBm (1GHz). Thus this saturation of output voltages limits the RF-DC conversion efficiency from reaching higher efficiency up to the perfect level. However, this investigation is still on the way and will be discussed elsewhere. The RF-to-DC conversion efficiency, *η* of the diode can be calculated using Equation (3) [6]:

(3) $$\eta =\frac{P_{\mathrm{out}}}{P_{\mathrm{in}}}\times 100\%$$

where *P_out_* is the DC power produced at the load, *R_L_* and *P_in_* is the injected power at input side of diode. Using Equation (3), we calculate the RF-to-DC conversion efficiency in terms of input power at 10 MHz, 50 MHz and 1 GHz.

**Figure 7.**
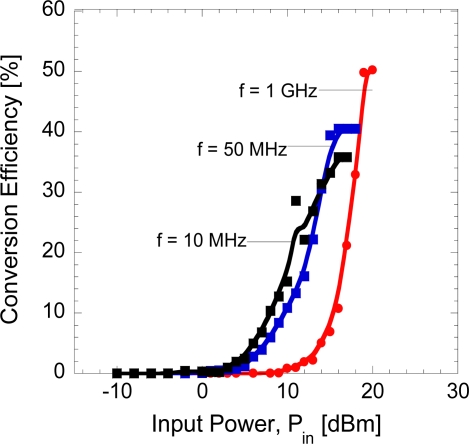
Conversion efficiency as a function of input power at frequency of 10 MHz, 50 MHz and 1 GHz.

Figure 7 shows the measured conversion efficiency of diode as a function of input power at different frequencies. Here, it can be seen that up to 50% conversion efficiency is obtained from RF-to-DC measurement at frequency of 1 GHz with series connection of diode and load. Detail modeling analyses to reveal the causes for the limitation of conversion are underway. However, an overview of the applied theoretical procedure is summarized here. Since the rectifying process is a nonlinear process, it is difficult to figure out how the rectifying circuit is optimized for the maximum conversion efficiency. There are several theoretical analyses to solve this problem. These analyses can be divided into two methods. One is to directly simulate the rectifying circuit in the time domain [36,37]. The other is to find a closed-form equation which can explain the relationship between diode parameters and the conversion efficiency [36,37,42]. A modified close-form equation for the conversion efficiency adapted from [37] has been chosen to analyze the conversion characteristics of the diode. In this analysis, we assume that the effects of harmonics higher than or equal to the second wave order are small and the forward voltage drop of the diode doesn’t change during the turn-on period. The diode conversion efficiency then depends only on the diode electrical parameters and the circuit losses at the fundamental frequency and dc. The maximum efficiency limited by the series resistance and the junction capacitance of a diode has been calculated using the modified closed-form equation. The breakdown voltage is not considered in the calculation since it is much larger than the junction voltage of the diode which is about 1.1 V. Therefore, ideally the efficiency can increase nearly 100% before reaching at the region where the breakdown effect is dominant. A mathematical model of the diode efficiency under those conditions has been derived to account for varying input power levels at any arbitrary frequency.

From another point of view, the RF-to-DC conversion efficiency can be improved by reducing the series resistance down to several Ω. At the moment, our device has series resistance of several kΩ. The reduction of series resistance can be achieved by removing the cap layer so that the ohmic contact can be formed directly on *n*-AlGaAs layer. The measurement of RF-DC conversion with parallel connection of diode and load will also be performed in future.

Table 1 summarizes the physical characteristics of AlGaAs/GaAs HEMT Schottky diode performed in this study and Si Schottky diode reported in [9].

The cutoff frequency, *f_c_* of the studied diode is 3 GHz which is one order lower than Si diode reported in [9]. However, the cut-off frequency can be increased if the Schottky contact area and contact distance are reduced as discussed in [3]. Therefore, the cut-off frequency of the studied AlGaAs/GaAs HEMT diode can be increased to be much higher than that of a Si diode if the dimensions of contact area and contact distance are reduced to the order of several μm. It can be seen that the SBH of the studied diode is almost close to the Si diode and can be further reduced by applying the metal with lower work function. The reverse leakage current of fabricated diode is also very small and also at the same level with the Si diode. From these characteristics, the AlGaAs/GaAs HEMT Schottky diode is a promising candidate to be used as an on-chip RF power detector as well as in low power rectenna devices.

## 5. Conclusions

In this paper, a preliminary investigation on the design, fabrication and characterization of a Schottky diode is performed. The increment ratio of output voltage *vs.* input power is 0.2 V/dBm, which is comparable to the 0.10–0.15 V/dBm of Si-based RF detector extracted from [9]. Conversion efficiency up to 50% is obtained from RF-to-DC measurement at 1 GHz. Low initial series resistance of the diode is needed to improve conversion efficiency. Investigation on metals with lower work functions such as Ti may become important so that lower barrier height can be realized since lower barrier height leads to better RF response. For a direct integration of planar antenna and Schottky diode via coplanar wave guide structure (CPW) without insertion of any matching circuit, the application of the same metals for the antenna and Schottky diode is preferable so that the impedance matching can be easily implemented and fabrication processes can be minimized or reduced. From our previous study on antennas as presented in [3] using Cr/Au or Ni/Au combinations, high return loss was obtained which promises good RF signal reception. Further studies on both devices (antenna and Schottky diode) using Ti and other metals should be explored. Further investigation of Schottky diodes with various chip contact sizes is also necessary in order to understand the dependence of RF power, cut-off frequency, *etc.* These experimental results will provide new breakthroughs for the direct on-chip integration technology towards realization of an RF detector as well as an ultra-low power rectenna technology to be integrated in nanosystems.

## Figures and Tables

**Table 1.** Physical characteristics of AlGaAs/GaAs HEMT Schottky diode and Si Schottky diode.

**Material**	**Area, *A* [μm^2^]**	**Distance, *d_diode_*[μm]**	**Schottky Contact**	***V_th_* [V]**	***R_s_* [Ω]**	***I_s_* [nA]**	**SBH[eV]**	***f_c_* [GHz]**	***C_j_* [fF]**	**Used equipments**
AlGaAs/GaAs (this work)	20 × 20	40	Ni/Au	1.1	1370	150	0.4349	3	39	Signal Generator Agilent 83650B Output: Tektronics TDS 3054C
Si (Ref. [9])	2 × 2	3	Al/n-Si	0.2	83	100	0.3890	12	160	Signal Generator Agilent 83731B Output: Tektronics TDS 620B
